# The receipt of psychological services and acceptability of local follow-up care for childhood cancer survivors: are there rural vs. urban differences?

**DOI:** 10.1007/s11764-025-01942-x

**Published:** 2026-01-08

**Authors:** Tyrone F. Borders, Abigayle R. Feather, Jerod L. Stapleton, John A. D’Orazio, Eric B. Durbin

**Affiliations:** 1 Department of Applied Health Sciences, Harris College of Nursing and Health Sciences, Texas Christian University, TCU Box 298615, Ft. Worth, TX 76129 USA; 2 Dept. of Psychology, College of Arts and Sciences, University of Kentucky, 111-E Kastle Hall, Lexington, KY 40536 USA; 3 College of Public Health, University of Kentucky, 111 Washington Ave, Lexington, KY 40536 USA; 4 Division of Hematology/Oncology, Department of Pediatrics, College of Medicine, University of Kentucky, Combs Cancer Research Building, 744 Rose St, Lexington, KY 40536-0096 USA; 5 Division of Biomedical Informatics, College of Medicine, Markey Cancer Center, Kentucky Cancer Registry, University of Kentucky, 2365 Harrodsburg Rd., Suite A230, Lexington, KY 40504-3381 USA

**Keywords:** Cancer survivor, Rural, Urban, Follow-up care, Children, Access

## Abstract

**Purpose:**

This study examined rural and urban parents’ reports of their children’s receipt of general follow-up care and psychological services, ratings of the acceptability of common sources of follow-up care, and ratings of local/nearby follow-up care access and effectiveness.

**Methods:**

A cross-sectional, quantitative survey was conducted among 238 (139 urban, 46 rural adjacent, and 53 rural non-adjacent) parents of childhood cancer survivors (ages 2–17 and at least 2 years since cancer diagnosis) recruited through the Kentucky Cancer Registry. Bivariate analyses and multivariable logistic regressions adjusting for predisposing, enabling, and need factors tested for differences across residence in urban counties, rural counties adjacent to an urban county, and rural counties non-adjacent to an urban county.

**Results:**

Children in rural adjacent counties had higher adjusted odds of receiving psychological services (OR 4.37; 95% CI 1.45, 13.22) than children in urban counties. Parents of children in non-adjacent counties had lower adjusted odds of agreeing that they could obtain local/nearby follow-up care for their child (OR 0.15; 95% CI 0.06, 0.36) and that local follow-up care was effective (OR 0.22; 95% CI 0.10, 0.48) than parents of children in urban counties.

**Conclusions:**

The study findings provide a comprehensive yet complex picture of similarities and differences in children’s follow-up care access according to their rurality of residence. Children residing in rural areas adjacent to an urban area have better access to psychological services than children residing in urban areas. However, parents of children residing in rural areas non-adjacent to an urban area have worse ratings of the accessibility and effectiveness of local follow-up care.

**Implications for Cancer Survivors:**

Psychological services may need to be expanded for childhood cancer survivors in both rural and urban areas to ensure equitable access. The accessibility and quality of local services may need to be improved to encourage the use of local follow-up care among children residing in rural areas that are non-adjacent to an urban area.

## Introduction

Clinical guidelines recommend that cancer survivors receive follow-up care to monitor for cancer recurrence, screen for the development of new cancers, and offer treatment for physical and psychological side effects [1, 2]. Childhood cancer survivors residing in rural areas, many of whom travel to urban-based cancer treatment centers, may face particular challenges transitioning to follow-up care when returning to their home communities. However, a paucity of research has examined childhood cancer survivors’ follow-up care access by rurality of residence. Many of the studies that have been conducted have focused on the adulthood period following childhood cancer, rather than the child or adolescent period following a cancer diagnosis. One study found that childhood cancer survivors who resided more than 100 miles away from an urban-based cancer center in Utah were more likely to visit its survivorship clinic than survivors who lived 15 or fewer miles away [3]. Other qualitative research conducted among rural adult survivors of childhood cancer in Utah has shown that many venture to urban areas for their follow-up care [4]. Concerns about distance to a specialty follow-up care center are further supported by patients treated at an Oklahoma cancer center, which found that those residing in large towns had a higher likelihood of receiving inadequate follow-up care than their urban counterparts [5].

Although the receipt of services from a survivorship clinic is associated with improved health outcomes [6], few survivorship clinics exist in rural areas nationally. Many rural childhood cancer survivors may therefore turn to primary care professionals (PCPs), especially family physicians, as they are the predominant physician providers in rural America [7]. However, questions remain about the acceptability of follow-up care delivered in primary care settings. Even though the majority of participants in a qualitative study reported having a primary care provider (PCP), many cited reservations about the quality of local primary care for addressing cancer-related concerns [4]. Some children may receive services through a hybrid model of highly specialized care delivered at pediatric cancer treatment centers in coordination with care delivered in community settings [8–10]. However, no prior research has examined the acceptability of PCP or hybrid follow-up care models among rural or urban parents.

A slightly larger body of relevant research has examined rural vs. urban differences in adult cancer survivors’ receipt and ratings of their follow-up care. In a qualitative study conducted in Alaska and Oregon, many rural adult survivors expressed challenges traveling to receive care [11]. Analyses of the Medical Expenditure Panel Survey (MEPS) revealed that lower percentages of rural, relative to urban, adult survivors discussed with their provider their cancer treatments [12]. Research based on Surveillance, Epidemiology, and End Results (SEER) program data linked to Consumer Assessment of Healthcare Providers and Systems (CAHSPS®) surveys indicated that rural adult cancer survivors had better care coordination relative to urban adult cancer survivors [13], but no other rural vs. urban differences [14]. Lastly, analyses of the Medicare Current Beneficiary Survey (MCBS) found that lower percentages of rural than urban cancer survivors were satisfied with the ease and convenience of seeing providers as well as their abilities to obtain all of their health care needs at a single site [15]. Although the findings from this body of research among adults are mixed, they point to at least some inequities in follow-up care access among rural adult cancer survivors.

This study’s primary purpose was to examine multiple dimensions of rural and urban children’s access to cancer follow-up care, including their receipt and sources of follow-up care, ratings of the accessibility and effectiveness of local follow-up care, and the acceptability of various sources of follow-up care. The study extends understanding of rural vs. urban similarities and differences in follow-up care accessibility, which may inform health care providers and health care systems in their efforts to implement services and programs to improve access for childhood cancer survivors.

## Methods

### Study design

A cross-sectional, quantitative survey was conducted among 238 parents or guardians (hereafter referred to as parents) of childhood cancer survivors. Children currently between the ages of 2 and 17 and at least 2 years from their cancer diagnosis were identified through the Kentucky Cancer Registry (KCR), a statewide population-based cancer registry that is part of the SEER program. KCR staff members also contacted the treating physician documented in the KCR to determine if there was any reason why the parent should not be contacted. A total of 698 children met these study inclusion criteria.

Parents of childhood cancer survivors were then recruited through a multi-step process involving KCR staff members and study personnel. First, KCR staff members mailed a letter addressed to the parent(s) of the identified child. The letter contained a description of the study, informed the parent of their eligibility based on their child’s age and cancer diagnosis, and included a pre-addressed card to return to the KCR granting permission for study team personnel to contact them. After initial mailings, KCR staff also made reminder phone calls to the parents of eligible children to maximize participation. Of the 698 eligible parents, KCR staff members were able to confirm contact and receive permission from 408 parents/guardians for study personnel to contact them to complete a survey. Reasons for not confirming contact included returned mail and disconnected telephone numbers. KCR staff then provided study personnel a list of 371 eligible parents/guardians who agreed to be contacted for participation in the survey.

Next, study staff members mailed a packet that included a study invitation letter, study consent information, a postcard that could be returned indicating that the individual declined to participate, and instructions about how to complete and return the survey via mail or the Research Electronic Data Capture (REDCap) web-based system [16, 17]. A reminder postcard was sent to parents who did not return a postcard indicating that they declined to participate and those who did not return a survey in either form within 30 days. A second reminder postcard was mailed within 30 more days after not receiving a response. Completion and return of the paper survey via mail or REDCap served as evidence of consent. After a survey was returned, study staff members emailed the participating parent a digital $50 gift card to compensate them for their time. A total of 238 parents completed a survey. Children residing in rural counties were oversampled to achieve an approximately equal number of non-metropolitan and metropolitan participants (rural *n* = 117, urban *n* = 121) based on U.S. Office of Management and Budget designations [18].

### Conceptual framework

The study was guided by the Behavioral Model of Health Services [19, 20], which posits how predisposing, enabling, and need factors influence health care access and has been used in research on adult cancer survivors’ health care utilization [21] and survivorship care needs [12]. Briefly, predisposing factors include demographic factors, such as age and gender, that serve as proxies for biological and cultural characteristics influencing an individual’s propensity to need and use health services. Enabling factors are posited to more directly influence an individual’s ability to obtain health services, such as whether they reside in a rural or urban community and their health insurance coverage. Lastly, need factors reflect an individual’s health status, such as their health-related quality of life and time since treatment.

### Dependent variables

The survey included a comprehensive array of measures to portray (1) the past 6-month receipt of follow-up care, (2) sources of follow-up care, (3) ratings of the accessibility and effectiveness of local follow-up care, and (4) ratings of the acceptability of various sources of follow-up care, all of which are described in more detail below.

#### Past 6-month receipt of follow-up care

Measures of the receipt of any follow-up care and follow-up care for psychological/counseling services in the past 6 months were “In the past 6 months, did your child see any physician, physician assistant, or nurse practitioners specifically for cancer follow-up care?” and “In the past 6 months, has your child received any psychological or counseling services related to the effects of cancer or cancer treatment?”.

#### Sources of follow-up care

Among those who received follow-up care in the past 6 months, additional items asked about sources of follow-up care or the health care provider that the child saw the most often (oncology, primary care, or other provider) and the location of the provider (community-based clinic/hospital or a university-based clinic).

#### Acceptability of follow-up care sources

Separate questions asked about the acceptability of several sources of follow-up care (university-based cancer center, university-affiliated cancer center, primary care providers, and primary care providers working with a cancer treatment center). Responses were on a 5-point scale ranging from 1 = very acceptable to 5 = not acceptable at all. Based on the distribution of responses, a dichotomous variable was created to indicate very acceptable/acceptable vs. neutral/not acceptable/not acceptable at all.

#### Ratings of the accessibility and effectiveness of follow-up care

Ratings of local access to follow-up care were assessed with two items developed for this study. The first stated, “A child who is in need of follow-up care for cancer can get it in or nearby my community.” The second stated, “Follow-up care for children’s cancer in or nearby my community is effective.” Response options ranged from 1 = strongly disagree to 5 = strongly agree. Based on the distribution of responses, variables representing strongly agree/agree vs. neutral/disagree/strongly disagree were created for each item.

### Independent variables

#### Predisposing factors

Child predisposing factors included age, race/ethnicity (based on the distribution of responses, this was coded as non-Hispanic White vs. other), and gender (we note that the survey included transgender and other gender options, but no parent selected those options).

#### Enabling factors

Child enabling factors included rural/urban residence and health insurance. For analysis, we further distinguished rural counties corresponding to Rural and Urban Continuum Codes as rural adjacent (codes 4, 6, and 8, or non-metropolitan counties adjacent to a metropolitan area) and rural non-adjacent (codes 5, 7, and 9, or non-metropolitan counties not adjacent to a metropolitan area) [22]. We note that we considered more discrete codes, but the cell sizes were small for the counseling/psychological services variable. Health insurance coverage was based on questions asking about whether the child was covered by Medicaid, Medicare, private, military, and/or other insurance (based on the distribution of responses, these were categorized as Medicaid, private, and other government insurance).

#### Need factors

Child need factors were time since cancer treatment ended (dummy variables representing not ended, < 1 year, 1 to < 3 years, and ≥ 3 years), number of symptoms (among a list of 12 symptoms including nausea, headaches, and changes in weight) stemming from cancer or cancer treatment, and quality of life (physical functioning and emotional distress scores assessed with an adapted version of the Pediatric Oncology Quality of Life Scale [23]). For the quality of life scale, participants were presented with statements such as “My child has had less energy and has been easily tired out” and selected responses on a Likert-type scale from 1 “never” to 7 “very frequently.” For each subscale, the items were summed to create physical functioning and emotional distress subscales, with higher scores reflecting greater impairment in each area [23].

### Analysis

Descriptive statistics were estimated for the sample characteristics and follow-up care measures. Chi-square tests were conducted to compare dichotomous dependent variables, and analyses of variance (ANOVA) were conducted to compare continuous variables across urban, rural adjacent, and rural non-adjacent residence. Among the dependent variables for which we found urban, rural adjacent, and rural non-adjacent differences in unadjusted analyses, we conducted multivariable logistic regression analyses to determine if those differences persisted while adjusting for predisposing, enabling, and need factors.

## Results

### Sample characteristics

Survey completion rates did not differ across the urban and rural residence categories. Of 218 urban parents, 63.76% completed a survey. Of 69 rural adjacent parents/guardians, 66.67% completed a survey; and of 84 rural non-adjacent parents, 63.10% completed a survey. Table 1 displays child predisposing, enabling, and need statistics by urban, rural adjacent, and rural non-adjacent residence. Non-Hispanic ethnicity varied significantly by residence, with higher proportions of rural adjacent (93.48%) and rural non-adjacent (88.68%) respondents being non-Hispanic white compared to urban children (77.70%). Insurance status also varied significantly by residence, with lower proportions of rural adjacent (26.09%) and rural non-adjacent (47.17%) children having private insurance compared to urban children (63.31%). Significantly higher proportions of rural adjacent (58.70%) and rural non-adjacent (52.83%) compared to urban children (35.97%) had Medicaid coverage. Lastly, physical functioning varied by residence, with rural non-adjacent having higher or worse physical functioning scores (mean 25.29) than rural adjacent (mean 20.88) and urban children (21.18).

**Table 1:** Predisposing, enabling, and need factors by urban and rural residence

Variable		Total sample (*N* = 238)	Urban (*N* = 139)	Rural adjacent (*N* = 46)	Rural non-adjacent (*N* = 53)	*P*-value
Predisposing	Non-Hispanic White, %	83.19	77.70	93.48	88.68	0.022
	Male, %	56.12	52.52	67.39	55.77	0.211
	Age, mean (SD)	11.08 (4.28)	11.08 (4.18)	11.48 (4.27)	10.73 (4.58)	0.696
Enabling	Private, %	52.52	63.31	26.09	47.17	< 0.0001
	Medicaid, %	44.12	35.97	58.70	52.83	0.009
	Other insurance, %	13.03	12.23	15.22	13.21	0.872
Need	Time since treatment ended					
	Not ended, %	11.34	10.79	15.22	9.43	0.889
	< 1 year, %	19.33	21.58	17.39	15.09	
	1 to < 3 years, %	44.96	43.17	43.48	50.94	
	3 or more years, %	24.37	24.46	23.91	24.53	
	Number of symptoms	1.80 (1.89)	1.64 (1.98)	1.96 (1.90)	2.08 (1.63)	0.299
	Physical functioning, mean (SD)	22.06 (10.13)	21.18 (9.91)	20.88 (9.89)	25.29 (10.41)	0.033
	Emotional distress, mean (SD)	18.03 (7.80)	17.60 (7.65)	18.27 (7.73)	18.96 (8.28)	0.547

*P*-value from chi-square or ANOVA

### Past 6-month receipt of follow-up care

**Fig. 1:**
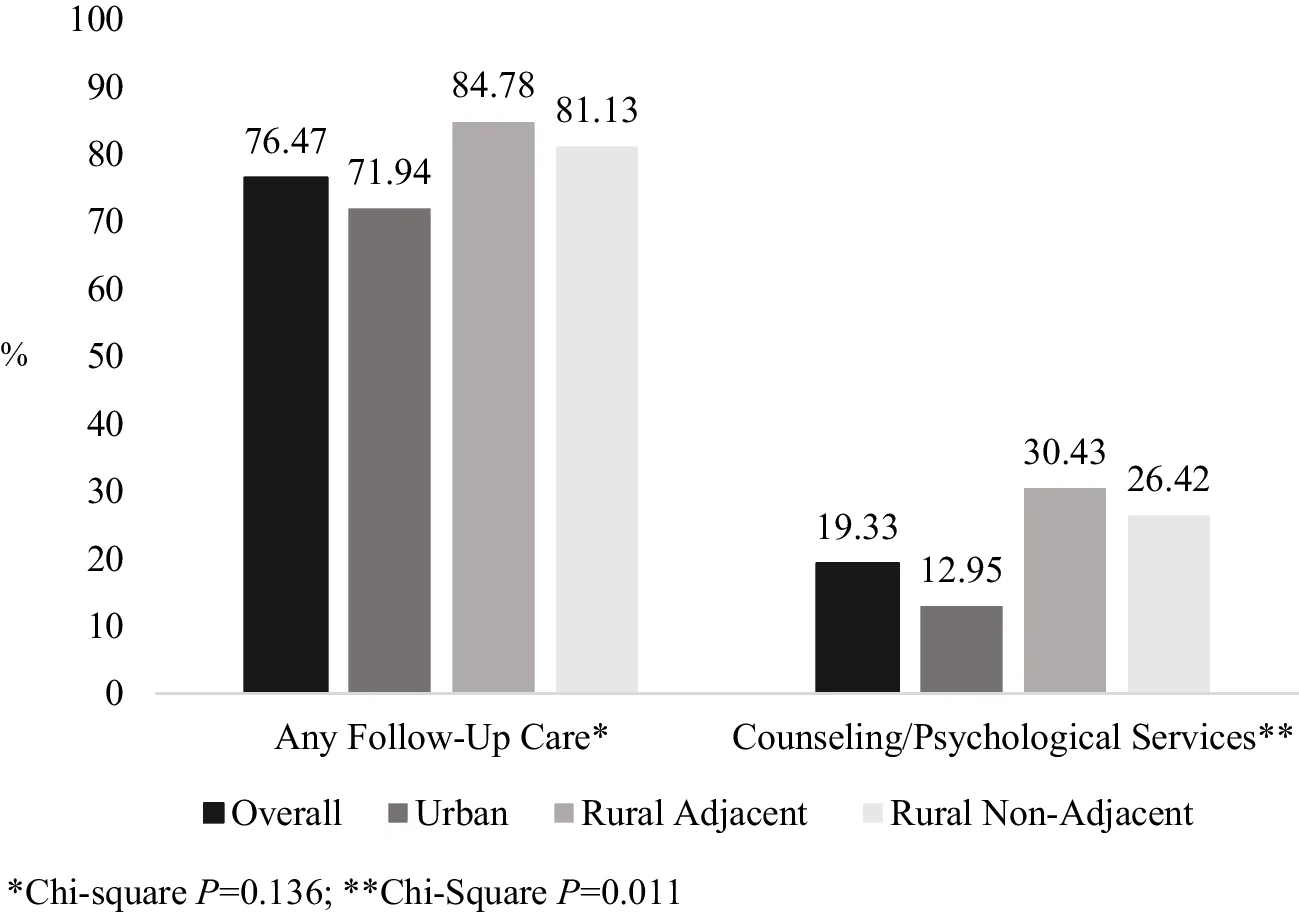
Receipt of follow-up care and psychological services in past 6 months

Figure 1 displays the receipt of any follow-up care and counseling/psychological services by residence. Similar proportions of urban (71.94%), rural adjacent (84.78%), and rural non-adjacent (81.13%) survivors received any type of follow-up care in the prior 6 months. The receipt of psychological/counseling services varied statistically significantly by residence, with lower proportions of urban (12.95%) than rural adjacent (30.43%) and rural non-adjacent (26.42%) receiving counseling/psychological services.

### Follow-up care sources, location, and acceptability

Table 2 shows descriptive statistics for the type of provider seen most often for follow-up care, the location/site of the provider seen most often for follow-up care, and ratings of the acceptability of common follow-up care sources, none of which varied statistically significantly by residence. The majority of children visited an oncologist (78.65%), and the majority saw a provider at a university-based hospital or clinic (77.88%). The vast majority of parents had positive ratings of the acceptability of university-based cancer centers for follow-up care (87.39%). Lower proportions of parents had positive ratings of the acceptability of follow-up care from university-affiliated cancer centers (62.18%), primary care providers (57.56%), and hybrid arrangements of PCPs working with cancer treatment centers (62.61%).

**Table 2:** Follow-up care sources, location, and acceptability by residence

		Total sample	Urban	Rural adjacent	Rural non-adjacent	*P*-value
Type of provider seen most often for follow-up care*	Oncology	78.65	78.35	74.36	83.33	0.766
	Primary care	9.55	8.25	12.82	9.52	
	Other	11.80	13.40	12.82	7.14	
Location of provider seen most often*	Community-based clinic/hospital	22.12	24.59	22.22	16.00	0.468
	University-based clinic/hospital	77.88	75.41	77.78	84.00	
Acceptability of follow-up care sources (very acceptable/acceptable)	University-based cancer center	87.39	85.61	86.95	92.45	0.440
	University-affiliated cancer center	62.18	62.59	54.35	67.92	0.378
	Primary care providers	57.56	56.12	63.04	56.60	0.703
	Hybrid PCP working with cancer treatment center	62.61	62.59	63.04	62.26	0.997

*Among those with past 6-month follow-up care

*P*-value from Chi-square or ANOVA

### Ratings of the accessibility and effectiveness of local follow-up care

**Fig. 2:**
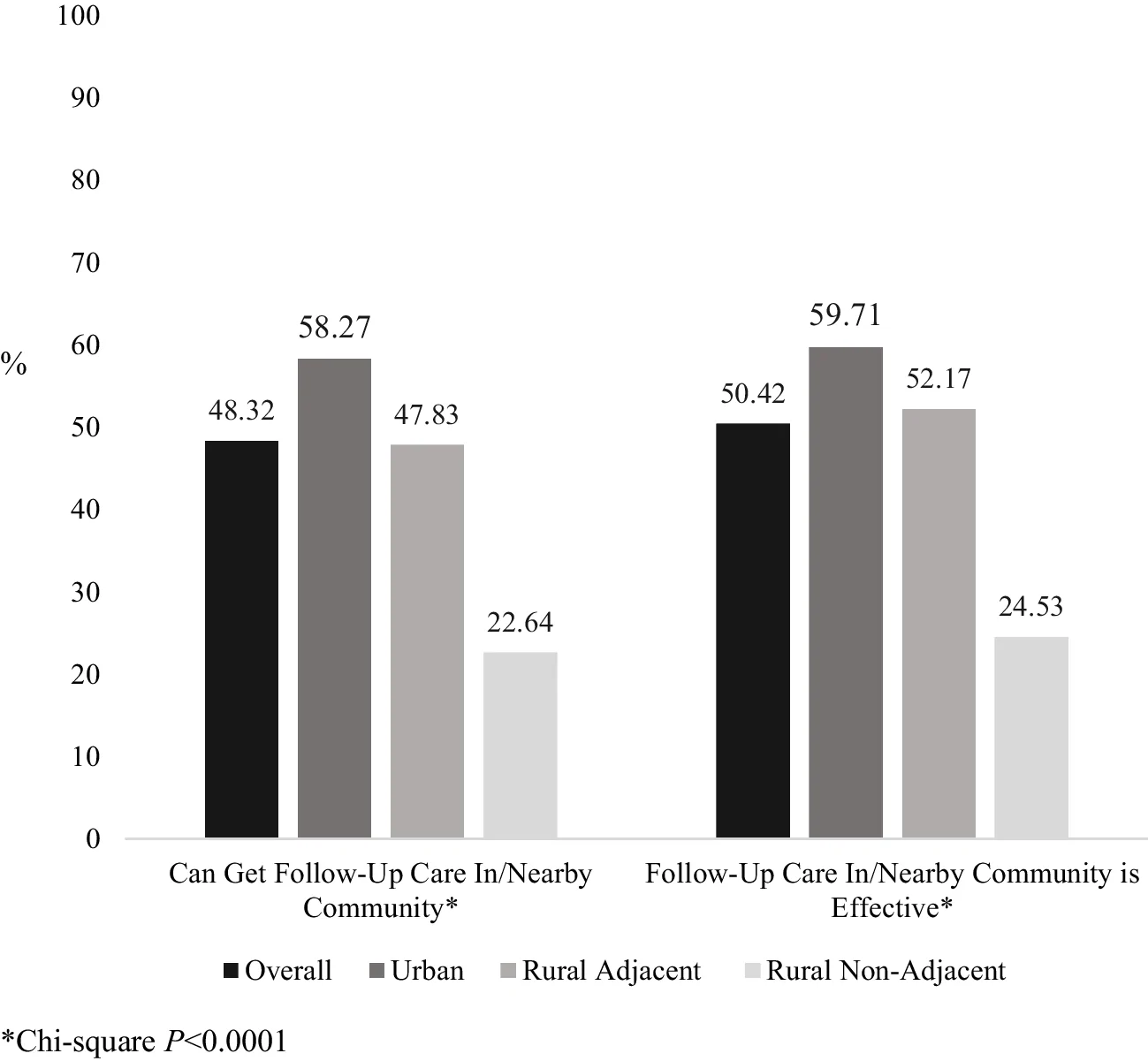
Strongly agree/agree that local/nearby follow-up care is accessible and effective

Figure 2 displays positive ratings of the accessibility and effectiveness of local follow-up care by residence. A minority of 47.83% of rural adjacent and only 22.64% of rural non-adjacent parents strongly agreed/agreed that they could obtain follow-up care for their child in/nearby their home community, compared to 58.27% of urban parents (*P* < 0.0001). Similarly, 52.17% of rural adjacent and only 24.53% of rural non-adjacent parents strongly agreed/agreed that follow-up care in/nearby their home community was effective, compared to 59.71% of urban parents (*P* < 0.0001).

### Multivariable logistic regression models

Findings from logistic regression analyses are presented in Table 3. Rural/urban variations in the receipt of counseling/psychological services and ratings of local/nearby follow-up care access and effectiveness persisted while adjusting for other factors. Rural adjacent, but not rural non-adjacent, children had higher adjusted odds of receiving counseling/psychological services (OR 4.37; 95% CI 1.45, 13.22) than urban children. Rural non-adjacent parents had lower adjusted odds of strongly agreeing/agreeing that they could obtain local/nearby follow-up care for their child (OR 0.15; 95% CI 0.06, 0.36) and strongly agreeing/agreeing that local follow-up care was effective (OR 0.22; 95% CI 0.10, 0.48) relative to urban parents. No other predisposing or enabling factors were predictors in any of the models. Of the need factors, only time since treatment was associated with the receipt of psychological services in the past 6 months. Children whose treatment had ended in the prior year had lower adjusted odds of receiving psychological services (OR 0.08; 95% CI 0.01, 0.62) than those whose treatment had not ended.

**Table 3:** Logistic regression models of receipt of psychological services and positive ratings of local follow-up care access and effectiveness

Variable		Received Psych. services in past 6 months	Strongly agree/agree can obtain follow-up care in community	Strongly agree/agree local follow-up care is effective
Predisposing	Rural adjacent (vs. urban)	**4.37** (1.45, 13.22)	0.77 (0.34, 1.75)	0.97 (0.43, 2.18)
	Rural non-adjacent (vs. urban)	2.33 (0.86, 6.34)	**0.15** (0.06, 0.36)	**0.22** (0.10, 0.48)
	Non-Hispanic White (vs. White)	2.04 (0.45, 9.26)	0.76 (0.32, 1.81)	0.47 (0.20, 1.11)
	Male (vs. female)	0.43 (0.18, 1.04)	0.71 (0.39, 1.32)	0.75 (0.41, 1.36)
	Age	0.98 (0.89, 1.09)	1.06 (0.98, 1.15)	1.08 (1.00, 1.17)
Enabling	Private (vs. no)	1.11 (0.29, 4.19)	2.25 (0.84, 6.02)	1.17 (0.45, 3.06)
	Medicaid (vs. no)	1.26 (0.35, 4.60)	1.18 (0.46, 3.02)	0.70 (0.28, 1.73)
	Other insurance (vs. no)	0.77 (0.17, 3.46)	0.75 (0.24, 2.42)	1.40 (0.45, 4.49)
Need	Time since treatment ended			
	< 1 year (vs. not ended)	**0.08** (0.01, 0.62)	0.34 (0.11, 1.08)	0.51 (0.16, 1.65)
	1 to < 3 years (vs. not ended)	0.40 (0.12, 1.40)	0.90 (0.32, 2.52)	0.92 (0.32, 2.62)
	≥ 3 years (vs. not ended)	1.10 (0.30, 3.99)	0.35 (0.11, 1.06)	0.35 (0.11, 1.09)
	Number of symptoms	1.15 (0.90, 1.48)	0.99 (0.81, 1.21)	1.02 (0.84, 1.25)
	Physical functioning	1.01 (0.96, 1.06)	0.99 (0.95, 1.02)	1.00 (0.97, 1.04)
	Emotional distress	1.14 (1.06, 1.22)	1.00 (0.95, 1.05)	1.00 (0.95, 1.05)

Significant *ORs* are in bold (*p* < 0.05)

## Discussion

The National Cancer Institute (NCI) has spotlighted research on rural/urban disparities overall given accumulating evidence of rural/urban disparities in cancer and treatment access [24–26]. Cancer survivors living in rural areas experience a higher cancer mortality rate and report poorer outcomes compared to survivors in urban areas [25, 26]. This study explored rural vs. urban variations in multiple dimensions of the utilization, accessibility, and acceptability of follow-up care services among a population-based sample of parents of childhood cancer survivors.

One strength of this analysis is the differentiation of children residing in rural areas according to their adjacency to an urban area. Even when adjusting for other factors, we found that children residing in a rural adjacent area had higher odds of receiving psychological services than urban children. This may be counterintuitive, but one potential explanation is that rural counties that are adjacent to an urban county have relatively strong economies and health care systems that support the availability of psychological services. Regardless of county population size, the study’s findings suggest a need to improve access to psychological services. A few studies have examined the use of telehealth in rural cancer care [27], which is a potential avenue of improving access to psychological services among rural and urban areas alike. One hurdle to expanding psychological services across the state is a shortage of mental health professionals, which exists in 111 of Kentucky’s 120 counties [28].

Although residence in a rural adjacent county was positively associated with the receipt of psychological services, residence in a rural non-adjacent county was negatively associated with positive ratings of being able to obtain local/nearby follow-up care and positive ratings of the effectiveness of local/nearby follow-up care. A possible justification for this finding is that such counties have few, if any, clinics offering follow-up care and expertise where parents are comfortable taking their children. Parents residing in rural non-adjacent areas may prefer to take their children to urban areas for follow-up care, which has implications for caseloads at urban-based clinics. In a qualitative study conducted in Utah, many rural adult survivors of childhood cancers expressed doubts about the quality of local primary care, which may have contributed to their readiness to drive to visit cancer specialty providers [4]. Although rural residents overall are more reliant on primary care than urban residents, we did not find this to be the case among childhood cancer survivors. Rather, the vast majority of both rural and urban survivors saw an oncology professional for follow-up care. A hybrid model in which PCPs work with a cancer treatment center may hold promise in terms of acceptability, cost savings, and effectiveness [29, 30]. In this study, approximately 63% of parents, regardless of residence, reported that a hybrid PCP/cancer treatment center model would be very acceptable/acceptable. One roadblock to expanding a hybrid model in Kentucky is a shortage of primary care professionals. Of Kentucky’s 120 counties, 118 have a primary care shortage [31].

### Limitations

The survey methods used in this study may have led to some biases in the findings. Parents’ reports of their children’s receipt of follow-up care services could be subject to recall bias, but any recall bias likely occurred among rural and urban parents alike. Studies based on insurance claims data would provide less biased estimates of the receipt of follow-up care, but such studies would exclude children without insurance coverage and would not provide any information about parents’ perceptions and ratings of various sources of potential follow-up care. Although the participation rate was high for a population-based study, the non-participants could have different perceptions of their children’s access. We did not find any rural/urban differences in the survey completion rates among parents who granted permission to the KCR for the study team to contact them, but we did not have information about the broader sample of parents (or their children) who did not grant such permission, which limits our ability to evaluate potential participation biases. Although we included a measure of emotional distress, we did not collect information about mental health diagnoses, which could better reflect the need for psychological follow-up services. Regarding external validity, the generalizability of the findings may be somewhat limited to Kentucky, but the findings could extend to other states with large rural populations. Overall, the study limitations are outweighed by its strengths, including sampling from a cancer registry.

## Conclusions

This paper provides a more comprehensive portrayal of similarities and differences in urban and rural parents’ reports of their children’s follow-up care access. Children residing in rural areas adjacent to an urban area actually had a higher likelihood of receiving psychological services than urban parents. Yet, parents of children residing in rural areas non-adjacent to an urban area had lower likelihoods of positive ratings of their abilities to obtain follow-up care locally and the effectiveness of local care relative to urban parents. To further understand the follow-up care preferences and decision-making among parents of childhood cancer survivors, and to improve children’s follow-up care access, we suggest that future research incorporate additional measures of trust in the oncology and primary care providers treating the child, the length of the relationships with those providers, and the degree of collaboration between oncology and primary care providers. Longitudinal study designs are also warranted to understand how these factors are associated with follow-up care access over time.

### Implications for cancer survivors

Psychological services may need to be expanded for childhood cancer survivors in both rural and urban areas to ensure equitable access. Ratings of local follow-up care services among parents residing in rural areas that are non-adjacent to an urban area suggest that the accessibility and quality of local services may need to be improved to encourage the use of local follow-up care.

## Data Availability

The data cannot be made available due to the presence of sensitive and confidential information.
